# Transcriptome analysis reveals non-identical microRNA profiles between arterial and venous plasma

**DOI:** 10.18632/oncotarget.15310

**Published:** 2017-02-14

**Authors:** Wenjing Xu, Yuan Zhou, Guoheng Xu, Bin Geng, Qinghua Cui

**Affiliations:** ^1^ School of Basic Medical Sciences, Peking University, Beijing, 100191, China

**Keywords:** circulating microRNA, arterial plasma, venous plasma, expression profile, microarray

## Abstract

Circulating microRNAs presented in venous plasma have been demonstrated as powerful biomarkers for the complex diseases like cancer. Nevertheless, those presented in arterial plasma remained largely unexplored. Here, using microarray technique, we compared microRNA expression profiles of the matched arterial and venous plasma samples from the same male rats. Though the microRNA profiles were largely similar, we identified 24 differentially expressed microRNAs, including 10 arterial highly expressed microRNAs and 14 venous highly expressed microRNAs. The differentially expressed microRNAs were validated by qRT-PCR. Computational analysis of these microRNAs and their targets indicated that arterial highly expressed microRNAs were overrepresented for functional terms like hematopoiesis and diseases like Crohn's Disease and leukemia; while venous highly expressed microRNAs were enriched for cell differentiation function, and diseases like distal myopathies and heart failure. Our analysis also suggested significant correlations between plasma microRNA expression and tissue microRNA expression. Four arterial highly expressed microRNAs also showed enriched expression in specific tissues and would be novel biomarker candidates.

## INTRODUCTION

MicroRNAs, as the core component of the post-transcriptional regulation machinery, play important roles in many biological processes like cell proliferation, differentiation, stress response and apoptosis [1]. Accumulating evidence has suggested the wide associations between deregulation of intracellular microRNAs and complex diseases like cancer, cardiovascular diseases and metabolic diseases [2–5]. Moreover, the microRNAs presented in the blood, also known as circulating microRNAs, were recently proposed to be promising biomarkers and targets for the disease diagnosis and therapy [6–9]. For example, elevated expression of miR-21 in venous plasma was observed in patients with non-small-cell lung carcinoma and gastric cancer [8]. By comparing the venous plasma microRNA expression profiles from patients with Takotsubo cardiomyopathy or acute myocardial infarction, miR-16 and miR-26a were found to be highly expressed in Takotsubo cardiomyopathy patients, while miR-1 and miR-133a were highly expressed in acute myocardial infarction patients. These microRNAs, when used in combination, were proven to be an accurate biomarker for discriminating these clinically indistinguishable, life threatening diseases [9]. Finally, by comprehensive profiling of blood microRNAs from 454 individuals, the deregulated circulating microRNAs across 14 disease situations (including but not limited to lung cancer, pancreatic ductal adenocarcinoma, melanoma) were screened and demonstrated [7].

**Figure 1:**
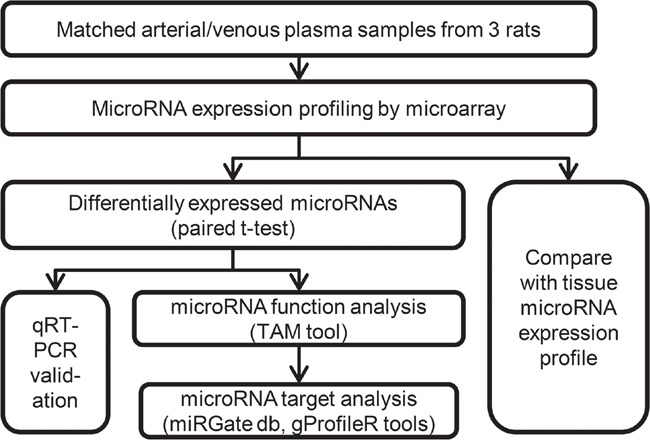
Flow chart depicting the pipeline of this study

However, it is noteworthy that most current circulating microRNA biomarkers were discovered in venous blood samples, and the arterial blood samples remained unexplored, partly due to 1) As for the clinical reality, the venous blood sample is used for most cases since it is safer and easier to obtain; 2) As for the biomedical researchers, there is an intuitive, yet non-validated assumption that the microRNA profiles in arterial and venous plasma should be identical. In this study, we examined whether microRNAs were uniformly expressed in matched arterial and venous plasma samples from three healthy male rats using microarray technique (Figure 1). Surprisingly, 24 differentially expressed microRNAs were identified between two groups of plasma samples. The function and disease associations of these microRNAs were analyzed. Finally, we found arterial expression profile of microRNAs exhibited significant positive correlation with tissue microRNA expression profiles, and such correlation was at least comparable to the correlation between venous microRNA expression profile and tissue microRNA expression profiles. MicroRNAs showing enriched expression in specific tissues were also presented in arterial plasma. These results indicate the potential of arterial plasma microRNAs as novel biomarkers for disease prevention and therapy.

## RESULTS

### Non-identical microRNA expression profiles between arterial and venous plasma

**Figure 2:**
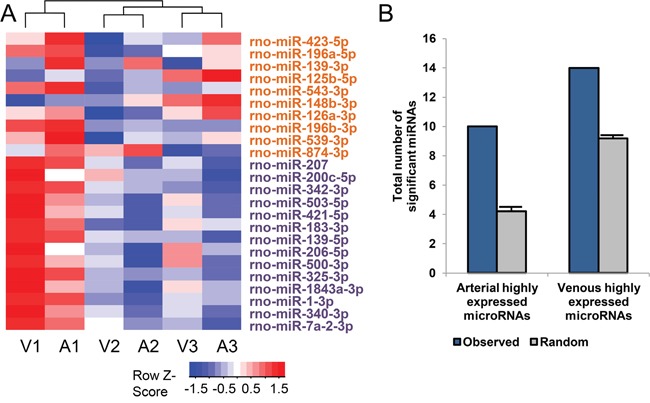
The 24 differentially expressed microRNAs between arterial and venous plasma **A**. Heatmap showing the expression pattern of 24 differentially expressed microRNAs. Samples were hierarchically clustered and the heatmap was scaled by rows (microRNAs). A1, A2, A3 indicate arterial plasma samples from three male rats, while V1, V2, V3 indicate matched venous plasma samples from the same rats. MicroRNAs highly expressed in arterial and venous plasma were labeled orange and violet, respectively. **B**. Comparison of the observed number of differentially expressed microRNAs and the number resulted from the randomized expression profiles. More specifically, 100 randomly shuffled profiles were tested by paired t-test to find if equivalent number of differentially expressed microRNAs could also be identified from these randomized expression profiles. Error bars indicate standard error.

We isolated plasma samples from three healthy male rats. For each rat, one arterial plasma sample and one venous plasma sample were obtained, and these matched samples were used for subsequent microarray analysis. Because the plasma samples were exactly matched, we adopted paired t-test and a P-value cutoff of 0.025 to identify differentially expressed microRNAs. In line with the intuitive assumption, the microRNA expression profiles in arterial and venous plasma samples were highly similar (Spearman correlation coefficient = 0.863, P-value < 1e-200). However, these profiles were not identical since 24 differentially expressed microRNAs were identified between two groups, including 10 arterial highly expressed microRNAs and 14 venous highly expressed microRNAs (Table 1). A heat map of these differentially expressed microRNAs is shown in Figure 2A. It could be observed that the matched samples from the same individual were clustered together, but the expression of these differentially expressed microRNAs exhibited consistent changes between two groups of plasma samples for all individuals. For example, rno-miR-139-3p was consistently up-regulated in arterial plasma compared with venous plasma, though the absolute expression level might vary between individuals (Table 1). To test whether the identified consistently changed microRNAs could be randomly expected, we shuffled the expression profile of each plasma sample and re-performed the paired t-test for 100 times. As shown in Figure 2B, the observed number of differentially expressed microRNAs was obviously higher than the number resulted from the randomized expression profiles (4 and 8 on average, for arterial and venous plasma samples, respectively). This result indicated that the observed differentially expressed microRNAs were non-random.

**Table 1:** Differentially expressed microRNAs between the arterial plasma and venous plasma

microRNA	Group	Fold Change*	P-value	Consistency**
rno-miR-423-5p	Arterial	1.180	0.002	Yes
rno-miR-196a-5p	Arterial	1.011	0.005	Yes
rno-miR-139-3p	Arterial	1.439	0.006	Yes
rno-miR-125b-5p	Arterial	1.103	0.007	Yes
rno-miR-543-3p	Arterial	1.048	0.007	Yes
rno-miR-148b-3p	Arterial	1.019	0.009	Yes
rno-miR-126a-3p	Arterial	1.070	0.016	Yes
rno-miR-196b-3p	Arterial	1.020	0.019	Yes
rno-miR-539-3p	Arterial	1.045	0.021	Yes
rno-miR-874-3p	Arterial	1.035	0.025	Yes
rno-miR-7a-2-3p	Venous	1.050	0.002	Yes
rno-miR-340-3p	Venous	1.060	0.002	Yes
rno-miR-1-3p	Venous	1.020	0.008	Yes
rno-miR-1843a-3p***	Venous	1.056	0.010	Yes
rno-miR-325-3p	Venous	1.044	0.012	Yes
rno-miR-500-3p	Venous	1.095	0.013	Yes
rno-miR-206-5p	Venous	1.032	0.015	Yes
rno-miR-139-5p	Venous	1.027	0.017	Yes
rno-miR-183-3p	Venous	1.019	0.017	Yes
rno-miR-421-5p	Venous	1.024	0.019	Yes
rno-miR-503-5p	Venous	1.043	0.020	Yes
rno-miR-342-3p	Venous	1.083	0.020	Yes
rno-miR-200c-5p	Venous	1.062	0.021	Yes
rno-miR-207	Venous	1.039	0.023	Yes

* MicroRNAs differentially expressed between two groups were identified by paired t-test with the P-value cutoff of 0.25. Differentially expressed microRNAs were sorted by P-value. For each microRNA, the fold change was calculated by comparing the mean normalized expression value from one group against that from another group.

** Whether this microRNA showed consistent higher expression in one plasma sample group compared with the matched samples from the other group.

*** This microRNA has no human homolog.

**Figure 3:**
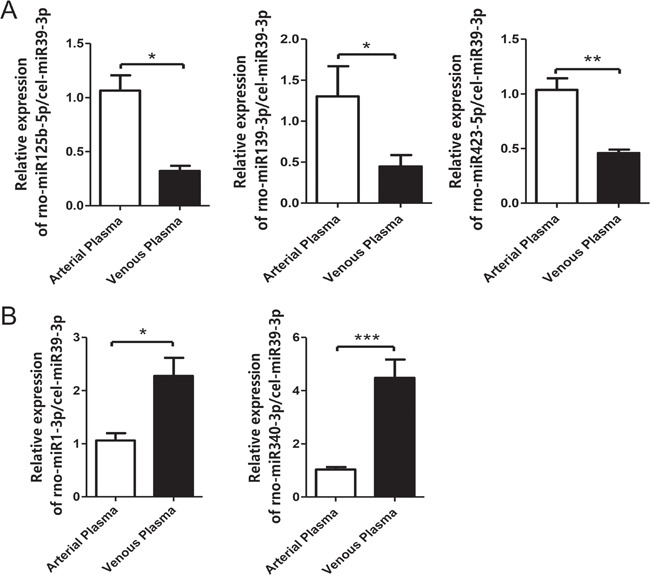
Validation of the differentially expressed microRNAs by qRT-PCR The *C. elegans* cel-miR-39-3p was used as the spike-in control. The results for arterial highly expressed microRNAs and venous highly expressed microRNAs were shown in panels **A**. and **B**., respectively. Error bars indicate standard error. Significance: * P-value <0.05, ** P-value <0.01, *** P-value <0.001.

Since there was non-negligible influence of individual variability on the plasma microRNA expression profiles, an orthogonal validation assay with increased sample size was naturally required. For doing so, we re-sampled arterial and venous plasma from eight healthy male rats, and examined the expression changes by qRT-PCR. Five differentially expressed microRNAs were randomly selected for validation, including three arterial highly expressed microRNAs (rno-miR-139-3p, rno-miR-423-5p, rno-miR-125b-5p) and two venous highly expressed microRNAs (rno-miR-1-3p, rno-miR-340-3p). All microRNAs exhibited significant differential expression according to the results of qRT-PCR assay, and the direction of expression changes was fully agreed with the observations from microarray analysis (Figure 3). These results validated the significant differentially expressed microRNAs, and thus non-identical microRNA expression profiles between arterial and venous plasma.

### Functional and disease associations of differentially expressed microRNAs

**Figure 4:**
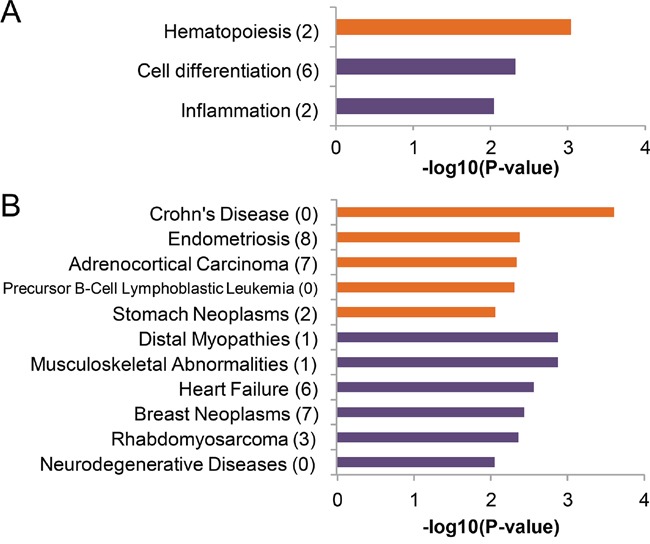
Enriched function and diseases of the differentially expressed microRNAs The nominal significant terms (P-value<0.01) with respect to **A**. function and **B**. diseases were listed here. The enriched terms of arterial highly expressed microRNAs and venous highly expressed microRNAs were indicated by orange and violet bars, respectively. We also tested if these terms could also be obtained from 1000 random set of equal number of microRNAs. The number of random sets showing association with the certain term was labeled in the parenthesis after the term name. Since no term was detected from more than 10 out of 1000 random sets, empirical P-values < 0.01 of all the listed terms were suggested.

We tried to explore the enriched functions and associated human diseases of the 24 differentially expressed microRNAs by TAM tool [3]. Unfortunately however, the default FDR cutoff did not result in any significant terms. One reason was that the functions of most differentially expressed microRNAs have not been well understood. Therefore, we re-performed the analysis with a loosed P-value cutoff of 0.01 (Figure 4). The results indicated that the arterial highly expressed microRNAs were overrepresented for hematopoiesis function (P-value = 9.15e-4), and the related disease term of precursor B-cell lymphoblastic leukemia-lymphoma (P-value = 4.96e-3). This association was mainly contributed by miR-125b and miR-126. The top enriched disease was Crohn' disease (P-value = 2.46e-4), which was contributed by miR-196a and miR-196b. The aforementioned four microRNAs also greatly contributed to the associations with other significant disease terms including endometriosis (P-value = 4.20e-3), adrenocortical carcinoma (P-value = 4.58e-3), stomach neoplasms (P-value = 4.96e-3), indicating that the function and disease association of most arterially highly expressed microRNAs awaits further experimental and clinical investigations. By contrast, the top enriched function terms for venous highly expressed microRNAs were cell differentiation. MiR-1 and miR-206 contributed the association with this term and related diseases like distal myopathies (P-value = 4.77e-3), musculoskeletal abnormalities (P-value = 1.34e-3) and rhabdomyosarcoma (P-value = 4.35e-3). Unlike the case of arterial highly expressed microRNAs, multiple microRNAs could contribute to the overrepresented terms. For example, seven microRNAs, including miR-1, miR-200c, miR-340, miR-342, miR-325, miR-139 and miR-500 contributed to the association with heart failure (P-value = 2.74e-3). This observation was in line with the fact that most previous studies of circulating microRNA were performed in venous samples, and therefore the function of venous highly expressed microRNAs were much better understood. Finally, we validated the significant terms by testing whether these terms could also be identified among 1000 sets of random microRNAs (each set contained 14 or 10 microRNAs, respectively) by using TAM tool with the same threshold. The total number of random sets associated with one significant term was labeled after the term name in Figure 4. We found the terms associated with the observed sets of differentially expressed microRNAs were rarely identified when testing random microRNA sets (0 to 8 times in 1000 random sets), indicating the significant terms in Figure 4 were not randomly expected (empirical P-value <0.01).

### Functional enrichment analysis of the targets of the differentially expressed microRNAs

**Figure 5:**
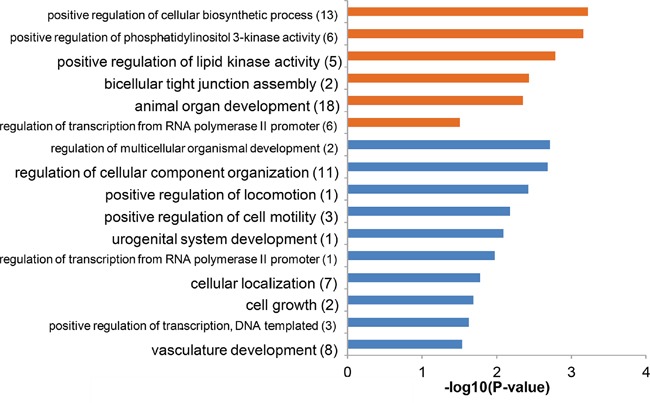
Enriched function of the targets of the differentially expressed microRNAs Representative significantly enriched terms (corrected P-value cutoff of 0.05, redundant terms removed by gProfileR) were listed here. The enriched terms of genes targeted by arterial highly expressed microRNAs only (i.e. arterial exclusive targets) were indicated by orange bars, while those of genes targeted by both arterial highly expressed microRNAs and venous highly expressed microRNAs (i.e. shared targets) were indicated by blue bars. We also tested if these terms could also be obtained from 1000 random set of equal number of targets. The number of random sets showing association with the certain term was labeled in the parenthesis after the term name. Since no term was detected from more than 20 out of 1000 random sets, empirical P-values < 0.02 of all the listed terms were suggested.

In above analysis, no overlapped significant terms were found between the two groups of differentially expressed microRNAs. This could be resulted from the limited knowledge about these microRNAs. To address this issue, we further analyzed the enriched function of the shared and exclusive targets of them. The high-confident targets (transcripts) were retrieved from the miRGate database [10], and 1957 arterial exclusive targets, 1232 venous exclusive targets and 333 shared targets were obtained. The enriched function of each group of microRNA targets were analyzed and clustered by gProfileR software package [11]. A corrected P-value (by Benjamini-Hochberg correction) of 0.05 was adopted for significant terms. Interestingly, we found the shared targets of arterial and venous highly expressed microRNAs were enriched in the cell development-related terms (Figure 5) like regulation of multicellular organismal development (corrected P-value = 1.94e-3) and cell growth (corrected P-value = 2.06e-2). Especially, the urogenital system development (corrected P-value = 8.13e-3) and vasculature development (corrected P-value = 2.92e-2) were enriched. For arterial exclusive targets, they were enriched not only enriched in cell development-related terms, but also in the positive regulation of lipid kinase activity (corrected P-value = 1.63e-3) and bicellular tight junction assembly (corrected P-value = 3.71e-3). We did not find specific terms associated with venous exclusive targets. When using DAVID 6.7 tool [12] instead, no significant enriched functional terms were detected for shared targets and venous exclusive targets. Analyzing all targets of venous highly expressed microRNA showed that their significant enriched terms were mostly covered by the terms associated with the shared targets. Finally, we noted that most of the enriched function terms listed in Figure 5 were general rather than specific. To test if these general terms could be detected by chance, for each group of targets, we assembled 1000 random target sets of the same size, and re-performed the functional enrichment analysis by gProfileR. The results indicated that the terms listed in Figure 5 could be identified in at most 18 out of 1000 random target set, suggesting empirical P-values < 0.02. Many terms could only be found in less than 10 ransom sets, indicating non-random term association with empirical P-values < 0.01.

### Comparison with tissue expression profiles of microRNAs

**Figure 6:**
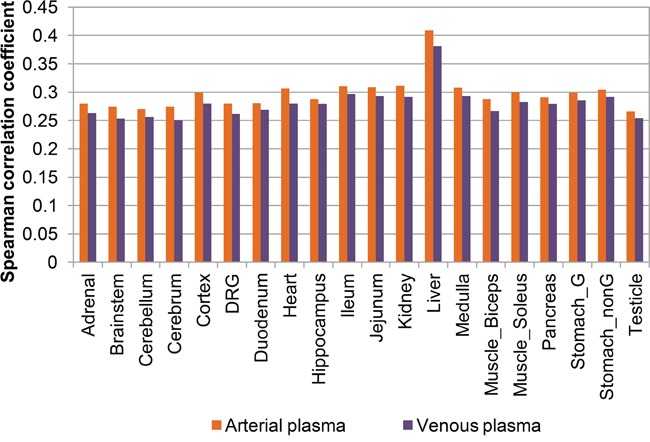
Expression correlation between plasma microRNAs and tissue microRNAs The correlation coefficient of arterial highly expressed microRNAs (orange bars) and venous highly expressed microRNAs (violet bars) with expression profiles of 20 different tissues were shown. Tissue abbreviations shown in this figure were: DRG, dorsal root ganglion; Stomach_G, glandular stomach; Stomach_nonG, non-glandular stomach.

An intriguing feature of circulating microRNA biomarkers is that their blood expression profiles are correlated with their expression profiles in tissues. Previous analysis of human microRNA expression profiles indicated that the venous blood expression was correlated with the tissue expression [6]. Here, we also compared the venous plasma microRNA expression profile measured by microarray here, with the microRNA expression profiles across 20 tissues measured by next-generation sequencing technique from Rat MicroRNA Body Atlas [13]. Interestingly, despite the technical differences, the venous plasma microRNA expression profile showed significant correlation with all of the tissue expression profiles (Spearman correlation coefficient > 0.25, and P-value of correlation < 1e-10; see also Figure 6). The venous plasma expression profile was most similar to that of liver, mimicking the previous analysis in human [6]. And expression profiles in several brain parts and the testicle exhibited lowest correlations, perhaps due to the existence of the blood-brain barrier and blood-testis barrier, respectively. We further analyzed the arterial plasma microRNA expression profile. As illustrated in Figure 6, the pattern of correlations was similar. Moreover, the arterial plasma microRNA expression profile showed slightly higher correlation with the tissue expression profiles.

Another concern is the specificity of the correlation between plasma microRNA expression and tissue microRNA expression. Though not all of the differentially expressed microRNAs identified in this study showed specific expression in certain tissues, several microRNAs with enriched expression in specific tissues could be found (Table 2). These microRNAs could serve as the promising candidates of circulating microRNA biomarkers for disease diagnosis and therapy.

**Table 2:** Enriched tissue expression of the differentially expressed microRNAs

microRNA	Group	Tissue with enriched expression	Related disease association*
rno-miR-196a-5p	Arterial	Muscle	-
rno-miR-539-3p	Arterial	Cerebrum, Hippocampus	Autistic disorder, Medulloblastoma
rno-miR-543-3p	Arterial	Cerebrum, Hippocampus, Brainstem	-
rno-miR-874-3p	Arterial	Cerebrum, Hippocampus	-
rno-miR-1a-3p	Venous	Muscle, Heart	Cardiac hypertrophy, Arrhythmogenesis
rno-miR-7a-2-3p	Venous	Hippocampus, Cerebrum	Autistic disorder, Schizophrenia, Neurilemmoma, Neurofibromatosis
rno-miR-325-3p	Venous	Cerebrum, Brainstem	-

* Only diseases related with the tissue where the microRNA exhibited enriched expression were listed. See discussion in main text for more detailed description about these microRNA-disease associations.

## DISCUSSION

Circulating non-coding RNAs (including microRNAs and long non-coding RNAs) have been increasingly noticed due to its stable existence in (venous) blood [7, 14], and its wide disease associations [2, 4, 8, 9]. Our analysis has demonstrated the non-identical microRNA expression profiles between arterial plasma and venous plasma. Interestingly, the arterial highly expressed microRNAs exhibited enriched functional terms and disease association that were not shared with venous highly expressed microRNAs (Figures 4 and 5). Moreover, the arterial plasma microRNA expression profile showed slightly higher correlation with tissue microRNA expression profiles (Figure 6). These observations together suggested novel biological importance of circulating microRNA presented in arterial plasma.

Our analysis also indicated intriguing correlation between plasma expression and specific tissue expression for several microRNAs (Table 2). These microRNAs have potential to be interesting biomarkers for disease diagnosis and therapy. Indeed, by searching against the well-established HMDD database [4], miR2Disease database [2] and recent literature, we found that a few of them have already been proposed to be associated with specific diseases. For example, miR-1 was a microRNA enriched in muscle and heart. It could relieve cardiomyocyte hypertrophy [15] and promote arrhythmogenesis in coronary artery disease [16]. And circulating miR-1 was demonstrated as a biomarker for cardiovascular diseases [17]. Another venous highly expressed microRNA miR-7 was enriched in nervous system. This microRNA was proposed to be associated with multiple nervous system disorders like autistic disorder [18] and schizophrenia [19], and act as tumor suppressor against schwannoma tumors [20]. Over-expression of miR-7 in venous plasma was also demonstrated as a biomarker of schizophrenia [21]. Likewise, the arterial highly expressed microRNA miR-539 showed elevated expression in the abnormal tissue in autistic disorder and medulloblastoma [18, 22]. But the capability of circulating miR-539 as the disease biomarker has not been tested. Moreover, for most arterial highly expressed microRNAs, their function and disease association await further investigation (Table 2).

In summary, our analysis indicated the potential significance of arterial circulating microRNAs. However, two important questions wait to be answered by future investigation. First, the casual factor of differential expression between arterial plasma and venous plasma remained elusive. Tissue release of microRNAs to the blood may play a critical role [6, 8], but the contribution of deregulated microRNAs in blood cells should also not be omitted. For instance, we noticed some hypoxia-responsive microRNAs [23] were differentially expressed. Such differential expression would be partly resulted from the altered blood cell microRNA expression profile in response to the altered oxygen content. Second, we only compared the microRNA expression profiles from healthy rats. To test the biomarker capability of arterial plasma microRNAs, their changes under disease conditions should be extensively investigated for the discovery of the novel biomarker.

## MATERIALS AND METHODS

### Ethics statement

Investigation has been conducted in accordance with the Declaration of Helsinki, Animal Management Rules of the Ministry of Health of the People's Republic of China and the guide for the Care and Use of the Laboratory Animals of Peking University. It has been approved by the authors' institutional review board. More specifically, male 10-week-old SD rats (280–300 g) were provided by the Animal Department, Health Science Center, Peking University. All the animals were housed in standard cages in a temperature- and humidity-controlled environment, on a 12 h light/dark cycle, with free access to water. All animal care and experimental protocols were approved by the Peking University Animal Ethics Committee.

### Plasma RNA isolation

Total 3mL of arterial or venous blood was collected from abdominal aorta or postcava respectively. And then blood was transferred into an anti-coagulation tube (EDTA, prepared with DEPC water) containing RNAase inhibitor. Plasma was obtained by centrifugation at 1600 g for 10 min, and the supernatant was centrifuged at 16000 g for 5 min at 4°C. Then the plasma was filtered through a 0.22-μm filter (MILLEXGV; Millipore). RNA was extracted using the miRNeasy Serum/Plasma kit (Qiagen; Valencia, CA) according to the manufacturer's protocol with modifications as recommended in previous studies [24, 25].

### Microarray analysis

The microRNA expression profiles from arterial or venous plasma samples were analyzed using the Agilent Rat miRNA V21.0 Microarray (SuperBiotek Corp, Shanghai, China) which covers 758 rat microRNAs. High quality microRNAs were Cy3 labeled using miRNA Complete Labeling and Hyb Kit (Agilent, USA) according to the manufacturer's guidelines. The microarray hybridization, washing and scanning were performed following the standard Agilent pipeline. Agilent Feature Extraction software (version 11.0.1.1) was used to analyze the acquired array images. Quantile normalization and subsequent data processing were performed using the GeneSpring GX v11.5.1 software package (Agilent Technologies). The differentially expressed microRNAs between arterial and venous plasma were compared using the paired t-test and P-value cutoff of 0.025. The entire datasets described here are available from the Gene Expression Omnibus (http://www.ncbi.nlm.nih.gov/geo/; accession number GSE90098).

### qRT-PCR assays

cDNA samples were prepared from total RNA of plasma. In total, 3 up-regulated and 2 down-regulated microRNAs in arterial plasma were analyzed by SYBR green I dye-based detection with specific primers. The relative expression of microRNA was determined by the 2^−ΔΔCt^ method with Spike-In Control (cel-miR-39-3p) expression to normalize the data. All the primers for rno-miR-423-5p, rno-miR-340-3p, rno-miR-139-3p, rno-miR-1-3p and rno-miR-125b-5p and *C. elegans* microRNA control cel-miR-39 were synthesized by RIBOBIO.

### microRNA functional and disease association analysis

The functional enrichment and disease association analysis of differentially expressed microRNAs were performed by using the TAM tool [3] with default parameters of TAM and version 2 of the annotation set. Because the default FDR threshold of TAM resulted in no significant terms, a P-value cutoff of 0.01 was applied instead. Note also that the rat microRNAs were mapped to their human orthologs before analysis since the TAM tool only support human microRNAs.

### Function enrichment analysis of the microRNA targets

We retrieved the microRNA target datasets from miRGate [10] database. For each differentially expressed microRNA, only the targets which were experimentally validated or supported by at least three independent predictions were retained. Targets exclusive to highly expressed microRNAs or venous highly expressed microRNAs, and those shared by these two groups of microRNAs were separately analyzed by gProfileR software package (v0.6.1) in R [11]. The Benjamini-Hochberg corrected P-value threshold of 0.05 was used to determine the significant functional terms. The significant functional terms were filtered using the ' Best Per Parent' criterion provided by gProfileR to avoid redundancy.

### Comparison with tissue microRNA expression profiles

The microRNA expression profiles across 20 tissues were obtained from Rat MicroRNA Body Atlas [13], and compared with the plasma microRNA expression profiles in this study by Spearman correlation coefficient. Note that only male rat tissue samples were considered. The tissue-enriched microRNAs were obtained from the same study.

## Abbreviations

qRT-PCR: real-time quantitative reverse transcription polymerase chain reaction
FDR: false discovery rate
